# Upregulation of ICAM-1 in diabetic rats after transient forebrain ischemia and reperfusion injury

**DOI:** 10.1186/s12950-014-0035-2

**Published:** 2014-11-05

**Authors:** Li Jing, Jian-Gang Wang, Jian-Zhong Zhang, Cai-Xia Cao, Yue Chang, Jian-Da Dong, Feng-Ying Guo, P Andy Li

**Affiliations:** Department of Pathology, Ningxia Medical University and Ningxia Key Laboratory for Cerebrocranial Diseases, Incubation Base of National Key Laboratory, Yinchuan, Ningxia P. R. China; Department of Pharmaceutical Sciences, Biomanufacturing Research Institute and Technological Enterprise (BRITE), North Carolina Central University, Durham, North Carolina USA

**Keywords:** Cerebral ischemia, Endothelia, Forebrain ischemia, ICAM-1, Inflammation, Neurodegeneration, Neuron, Stroke

## Abstract

**Background:**

Hyperglycemia exacerbates brain damage caused by cerebral ischemia. Neuroinflammation may play a role in mediating such enhanced damage. The objectives of this study were to examine the mRNA and protein levels and cell type distribution of ICAM-1 after cerebral ischemia in normo-and diabetic hyperglycemic rats.

**Results:**

Compared to normoglycemic ischemia animals, diabetes aggravated neuronal death, decreased Nissl body staining, and increased ICAM-1 mRNA and protein levels in the frontal cortex. The increased ICAM-1 was located not only in vascular endothelial cells but also in cortical neurons.

**Conclusions:**

Our results suggest that exacerbated neuro-inflammation in the brain may mediate the detrimental effects of diabetes on the ischemic brain.

## Background

It has been well established that preischemic hyperglycemia exacerbates ischemia induced neuronal damage and augments the infarct size [1,2]. The exact mechanisms of diabetic hyperglycemia exacerbating ischemia are not fully understood. However, studies have shown that hyperglycemia produces lactic acid, increases free radical formation, activates the intrinsic cell death pathway, disrupts the blood– brain barrier (BBB), and induces local inflammation [3].

Recent studies suggest that inflammation plays an important role in the occurrence and development of ischemic brain damage [2]. The infiltration of polymorphonuclear leukocyte (PMN) into ischemic brain tissue has been demonstrated in several animal models of stroke [4]. Cerebral ischemia results in vascular endothelial cell injury, leading to the expression of cell adhesion molecules and attracting PMN to attach to endothelial cells, which then migrate to ischemic tissue where they: release pro-inflammatory cytokines, produce free radicals and activate microglia [5]. The recruitment of circulating PMNs into ischemic tissue initially requires the interaction of microvascular endothelial cells with these inflammatory cells via specific adhesion molecules [6]. The expression of cell adhesion molecules and release of inflammatory cytokines are early events after ischemia and reperfusion. Several cell adhesion molecules are known to play critical roles in the cell surface interactions between endothelial cells and PMNs. One of the best-known adhesion molecules found on the endothelial cell surface is intercellular cell adhesion molecule 1 (ICAM-1) [6]. ICAM-1 is a glycoprotein that is expressed on the membrane of the vascular endothelium and other cells. It is upregulated by endotoxin and several cytokines including TNF-α, IL-1 and IFN-γ both *in vitro* and *in vivo* [7–9]. In addition to the activation of endothelial cells and trans-endothelial migration of PMN, the expression of ICAM-1 plays an important role in inducing neuro-inflammation and mediating the progression of ischemic injury after acute stroke [2]. Prevention of ICAM-1 expression by antisense infusion significantly decreases infarct size and the neurological deficits caused by transient focal ischemia [10,11].

Diabetic patients have an increased risk of developing cardiovascular disease and inflammatory microvascular complications in organs such as blood vessels, brain, heart, kidneys and retina. Hyperglycemia, the hallmark of diabetes, initiates macro- and microvascular complications in part by inducing an endothelial inflammatory phenotype [12]. Hyperglycemia upregulates cell adhesion molecules expressed on the vascular endothelium, a process known to induce pathological leukocyte-endothelium interactions. Recent evidence suggests that ICAM-1 not only promotes atherogenesis [13] but also exacerbates organ damage [12,14]. Although we and others have previously observed that ICAM and other pro-inflammatory cytokines are increased in diabetic animals after cerebral ischemia and reperfusion [15,16], these finding were confined to blood vessels and short period of reperfusion phases. The objective of this study was to characterize the ICAM expression pattern and its cellular localization after an extended period of reperfusion in both normo- and hyperglycemic ischemic animals.

## Methods

### Animals and reagents

Male Sprague-Dewley rats with body weights of 240-350 g were provided by the Medical Experiment Animal Center of Ningxia Medical University. All animal usage and procedures were in strict accordance with the Chinese Laboratory Animal Use Regulations. Efforts were made to minimize animal stress and to reduce the number of rats used for this study.

Polyclonal anti-GFAP antibody (Santa Cruz), monoclonal anti-NeuN antibody (Sigma), polyclonal anti-ICAM-1 antibody (Protect), polyclonal anti-β-actin antibody (Sigma), horseradish peroxidase-conjugated anti-mouse secondary antibody (Sigma), and streptozotocin (STZ, Calbiochem, Germany) and the ICAM-1 *In Situ* Hybridization Detection kit were purchased from Boster Biotechnology Co (Wuhan, China).

### STZ-induced diabetic hyperglycemia

The rats were injected intraperitoneally with streptozotocin (STZ, 55 mg/kg, in 0.1 mol/l citrate buffered saline, pH 4.5). Age-matched rats receiving the same volume of citrate-buffered saline served as normoglycemic controls. Blood glucose levels were measured 2–3 days after STZ injection to verify the success of diabetes induction. Those with glucose > 16 mmol/L were included in diabetic group. Cerebral ischemia was induced 7 days later in the STZ-induced diabetic and citrate buffer-injected non-diabetic animals.

### Experimental groups

Rats were randomly divided into three groups: (1) a sham-operated control group consisting of a normoglycemic and a hyperglycemic subgroup (n = 10); (2) a normoglycemic ischemic group (n = 20); and (3) a diabetic ischemic group (n = 20). The animals in the two ischemic groups (groups 2 and 3) were further divided into 4 sub-groups, namely 8 minutes of ischemia with 1-, 3-, and 6-days of reperfusion (n = 5 in each subgroup).

### Ischemic model

Both diabetic hyperglycemic and non-diabetic normoglycemic animals were subjected to an 8-min duration of forebrain ischemia induced by bilateral clamping of the common carotid arteries after exsanguinations from a femoral artery, maintaining blood pressure at 40–50 mmHg [17]. Brain ischemia was confirmed by an isoelectric EEG. The rats were revived after re-infusing the shed blood and releasing the ligatures placed around the carotid arteries. At the pre-determined time points, animals were euthanized, and their brains were removed. The brains were divided into left and right hemispheres. One half of the hemisphere was fixed in 4% paraformaldehyde buffer, processed and embedded in paraffin, and then sectioned at 5 μm intervals for histology and immunohistochemistry studies. The second half of the hemisphere was used for extraction of RNA and protein.

### Pischingert staining

After 10 minutes of incubation at room temperature in methylene blue solution, the sections were washed in PBS (pH 4.6) until the Nissl bodies were clearly visualized. The sections were then incubated with 4% ammonium molybdate buffer for 5 minutes. Neurons with Nissl body staining intensity decreased to less than 50% of the intensity of the control animals were defined as degenerative neurons.

### Real-time quantitative PCR

Total RNA was extracted using TRIzol (Invitrogen, Carlsbad, California, USA,) according to the manufacturer’s protocol. The total RNA concentration was assessed by measuring the absorbance at 260 nm using a Nano Drop Spectrophotometer (ND-1000, Thermo Scientific, USA). Reverse transcription (RT) for synthesizing the first-strand of the cDNA was performed with 2 μg of total RNA treated with M-MLV reverse transcriptase according to the manufacturer’s recommendations (Promega, USA). The resulting cDNA was then subjected to real-time quantitative PCR for evaluation of the relative mRNA levels of ICAM-1 and β-actin (as an internal control). Gene-specific amplification was performed using an ABI 7900HT real-time PCR system (Life Technologies, Carlsbad, California, USA) with a 15-μl PCR mix containing 0.5 μl of cDNA, 7.5 μl of 2× SYBR Green master mix (Invitrogen, Carlsbad, California, USA), and 200 nM of the appropriate oligonucleotide primers. The mix was preheated at 95°C (10 min) and then amplified at 95°C (30 sec) and 60°C (1 min) for 40 cycles. The Ct (threshold cycle) value of each sample was calculated from the threshold cycles using the instrument’s software (SDS 2.3), and the relative expression of the ICAM-1 mRNA was normalized to the β-actin value. The Ct values (number of the threshold cycle) of each sample were recorded. The difference in ICAM–1 gene expression was compared by varying the rate. The rate = 2^-ΔCt^ was varied, where ΔCt = (the Ct values of ICAM-1 in experimental group - the Ct values of β-actin in experimental group) - (the Ct values of ICAM-1 in sham control group-the Ct values of β-actin in sham control group). A relative expression change of more than 40% was considered significantly different. Beta-actin mRNA was used as a control for ICAM-1 mRNA, and normal rats were used as negative controls.

### Western blot analysis

Western blotting was employed to test whether upregulation of ICAM-1 mRNA led to an increase in ICAM-1 protein translation in the hyperglycemic animals. Western blotting was performed as previously described [18]. Brain tissues were homogenized using lysis buffer, and protein concentrations were measured using the Microplate BCA Protein Assay kit (Thermo Scientific). Equal amounts of protein (5 μg) were loaded on a 10% SDS-polyacrylamide gel and transferred to nitrocellulose membrane for 1 hour at 100 Volt. After being blocked with 5% non-fat milk, the membranes were incubated for 24 hours (4°C) using an anti-ICAM-1antibody (1:1000 dilution). Immunoreactivity was visualized using a Western blotting detection kit. Protein bands were scanned, and the optical density (OD) was measured using a computer imaging analysis system (Bio-Rad Co.).

### *In Situ* hybridization

Digoxigenin-labeled riboprobes, synthesized by Wuhan Boster Biological Engineering Limited (Wuhan China), were used for the detection of rat ICAM-1 and β-actin mRNA. The oligonucleotide probe sequences are as follows:

ICAM sense: 5’-TCACCTGGACAAGAAGGACTG-3’and

ICAM antisense: 5’-GTCAGATTAGGGGCTGGATTC-3’.

β-actin sense: 5’-TTTAATGTCACGCACGATTTC-3’and

β-actin antisense: 5’-CCCATCTATGAGGGTTACGC-3’.

The probe specificity was confirmed by BLAST software testing.

*In situ* hybridization was performed as previously reported [19]. Briefly, after regular sequence treatment, the sections were incubated with 20 μl of pre-hybridization solution for 3 hours (38-42°C). The sections were incubated with sense or anti-sense (control) riboprobe hybridization solution overnight (38-42°C). The reaction was stopped by applying blocking solution on the sections. Biotinylated mouse anti-digoxigenin was applied on the sections for 60 min (37°C), and followed by reaction with SABC. Biotin-peroxidase complex was applied, and diaminobezidine (DAB) was used as a substrate. The sections were counterstained with hematoxylin and mounted. The images were analyzed under a light microscope and are presented as a ratio of positively stained cells to total cells for an area of 50 mm^2^. Brown particles in the cytoplasm were considered a positive signal.

### Immunohistochemistry

Sections were treated with 3% H_2_O_2_ for 10 min at room temperature to quench endogenous peroxidase activity. Antigen retrival was achieved by heating sections immersed in sodium citrate buffer (pH 6.0) for 10 min using a microwave oven. The sections were incubated overnight (4°C) with primary antibodies against GFAP at 1:800 and ICAM-1 at 1:100. The sections were washed and then incubated with a horseradish peroxidase (HRP) conjugated secondary antibody (1:300, Santa Cruz). Color reaction was achieved by DAB incubation for 3 min at room temperature. The sections were then rinsed in distilled water and counterstained with hematoxylin. The sections were cover-slipped and analyzed using a computer image analysis system (Zeiss LSM5 Image Examiner software).

The immunofluorescent double labeling of ICAM-1 with the astrocyte marker GFAP or with the neuronal marker NeuN was also performed using the afore-mentioned antibodies. The double labeling of ICAM-1 and GFAP or ICAM-1 and NeuN was achieved by incubating with the two primary antibodies separately and then with a mixture of the two fluorescence-conjugated secondary antibodies. The specimens were mounted with Vectashield Hardset Mounting Media (H-1200, Vector) containing 4′, 6-diamidino-2-phenylindole (DAPI) and examined using a fluorescence confocal-scanning microscope (Nikon Eclipse C1).

### Statistical analysis

The values are expressed as the mean ± SEM. ANOVA followed by Tukey’s test was employed for statistical analysis among the three groups, and Student’s t-test was used to analyze data between two groups. P < 0.05 was considered to be statistically significant.

## Results

### Blood glucose alterations in rats

Diabetes was induced in the animals through streptozotocin injection (STZ, 55 mg/kg). The diabetic condition was confirmed in the rats through measurements of blood glucose levels 2 days after the STZ injection. Fasting blood glucose levels were measured again 10 min before the induction of cerebral ischemia. The mean blood glucose values were 5.72 ± 0.71 mmol/L for the normoglycemic animals and 19.98 ± 2.32 mmol/L for the diabetic animals (P < 0.01).

### Histological changes

**Figure 1:**
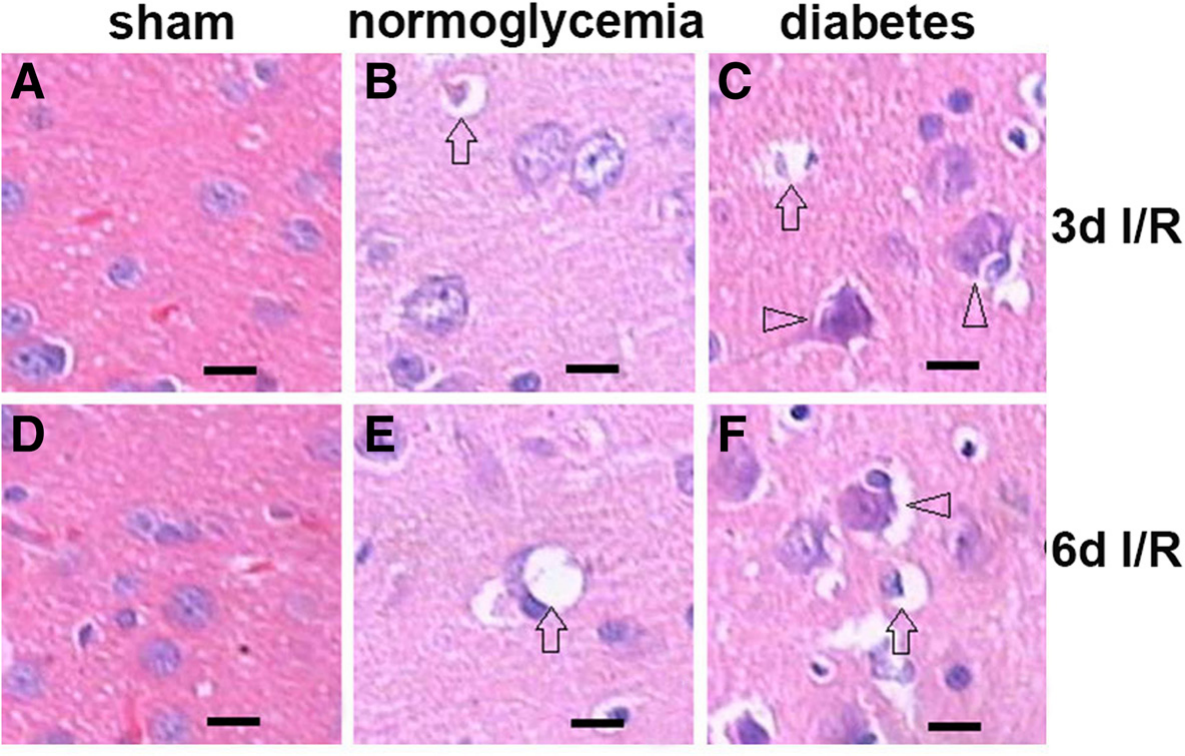
**Brain morphological changes in the frontal cortex after 8 min of cerebral forebrain ischemia and reperfusion in normoglycemic and hyperglycemic animals. (A,D)** Typical appearance of brain tissues in normo- and hyperglycemic sham control animals. **(B,E)** Brain edema and neuronal swelling in normoglycemic ischemic animals after 3 and 6 days of reperfusion. **(C,F)** Enhanced brain edema and neuronal death in hyperglycemic animals after 3 and 6 days of reperfusion. Arrows indicate tissue or neuronal swelling; arrowheads denote neuronal death. Magnification 400X, bar = 20 μm.

In this study, morphologic changes of the cerebral frontal cortex were detected using hematoxylin and eosin (H&E). As expected, there were no obvious cerebral structural abnormalities observed in the sham-operated normoglycemic and diabetic animals (Figure 1A and D). In the normoglycemic animals, transient forebrain ischemia for 8 min induced a mild brain edema at 1-day of reperfusion, which was identified by the increased swelling of the neuronal cell bodies and nucleus, and the widened intercellular space of the neurons, astrocytes and endothelial cells. At 3-day reperfusion, the brain edema was further increased. In addition, cellular swelling and the disappearance of the nucleolus were observed in a few neurons in the bilateral cerebral frontal cortex (Figure 1B). At this time point (3 days), a reduced number of neurons was observed. Some neurons showed abnormal cytoplasmic changes with pale staining, vacuolar degeneration, or dissolved nuclei. Although the brain edema was extenuated by 6 days of reperfusion, the neuronal damage had progressed significantly (Figure 1E). In the diabetic ischemic animals, the brain edema was clearly aggravated, and a more extensive loss of neurons and glial cells was observed compared to the normoglycemic ischemic group (Figure 1C). At 6 days of reperfusion, a large number of neural cells exhibited a shrunken triangular pyramidal cell body and pyknosis with deep staining (Figure 1F), suggesting these cells were undergoing degeneration or death.

### Neuro-degeneration detected by Pischingert staining

**Figure 2:**
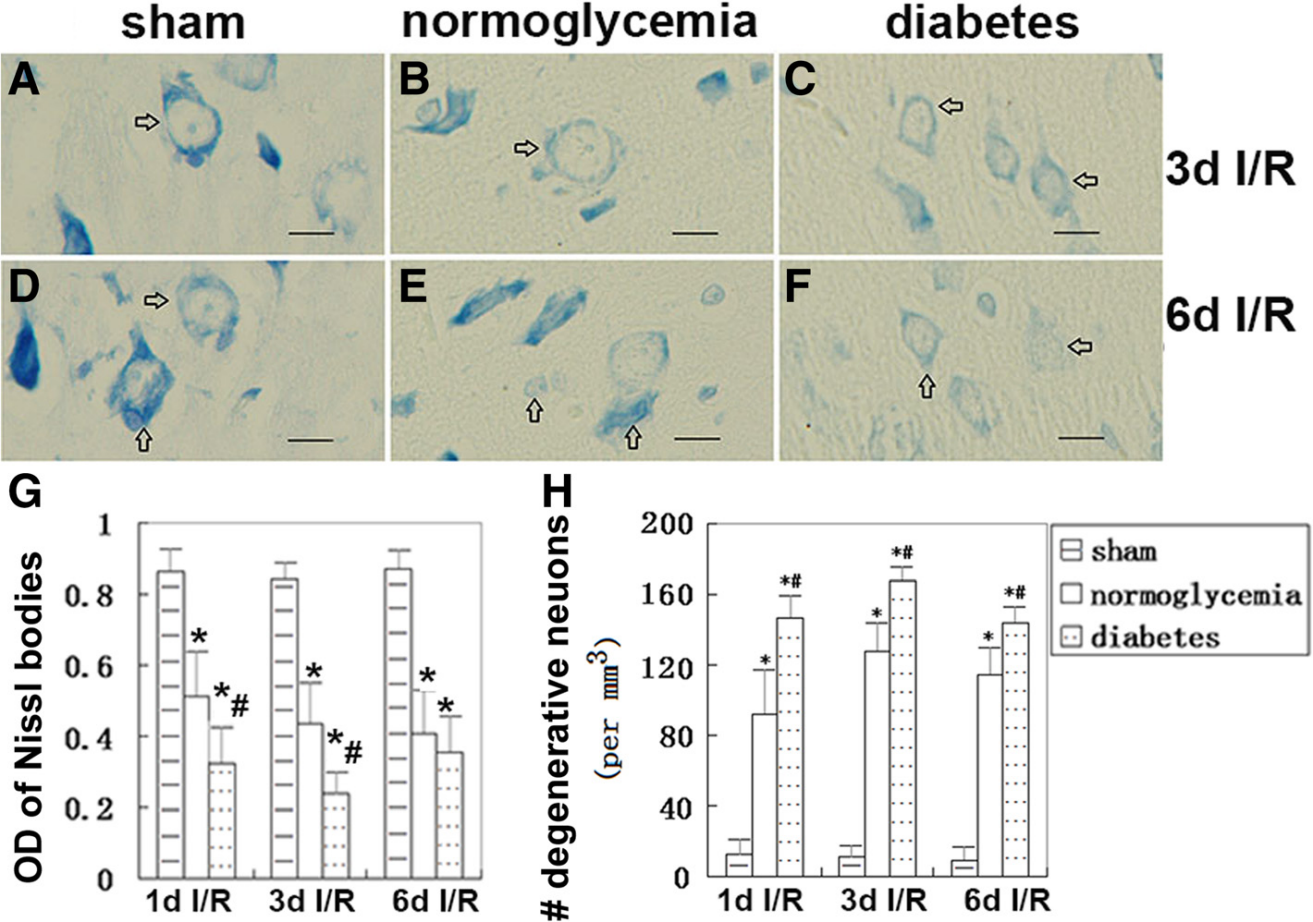
**Pischingert staining of the Nissl bodies in neurons of the frontal cortex after ischemia and reperfusion in normo- and hyperglycemic ischemic rats. (A,D)** Normal Nissl substances were stained in the cytoplasm of sham control animals. **(B,E)** Decreased staining intensity observed in normoglycemic ischemia rats. **(C,F)** Significantly weaker staining of Nissl substances in hyperglycemic ischemic rats. **(G)** Bar graph indicating the mean optical density (OD) of the Nissl bodies in the three groups at different reperfusion times. **(H)** The number of degenerative neurons in the cerebral frontal cortex. Arrows indicate positive Pischingert staining of the Nissl bodies. *P < 0.05 *vs*. sham group, #*P* < 0.05 *vs*. normoglycemic group at an identical reperfusion stage. Magnification, 400X, bar = 20 μm.

The Nissl bodies of neurons were stained by Pischingert and appeared as deep blue particles. The reduction or absence of Nissl bodies suggests the hydropic degeneration of neurons. The granular blue staining of the Nissl substance was uniformly detected in the cytoplasm of the frontal cortex of the sham-operated normoglycemic and diabetic hyperglycemic animals (Figure 2A and D). In the normoglycemic ischemic rats, the mean optical density [integral optical density (IOD)/Sum area] of the Pischingert staining was significantly decreased in the frontal cortex after 1, 3 and 6 days of reperfusion (Figure 2B, E and G). In comparison, diabetic forebrain ischemia induced a further decreased or even dissolved staining of the Nissl bodies (Figure 2C and F). As a result, the mean optical density of the Pischingert staining was significantly lower compared to the normoglycemic ischemic animals, especially at 1 and 3 days of reperfusion (Figure 2G). The numbers of degenerative neurons, as defined by a greater than 50% reduction of Nissl body staining, were counted in all three groups at 1, 3 and 6 days of reperfusion. Eight minutes of forebrain ischemia resulted in neurodegeneration in the frontal cortex from 1–6 days of reperfusion. Preischemic hyperglycemia further increased the number of degenerative neurons compared to the normoglycemic ischemic animals at an identical reperfusion stage (Figure 2H).

### ICAM-1 mRNA level

**Figure 3:**
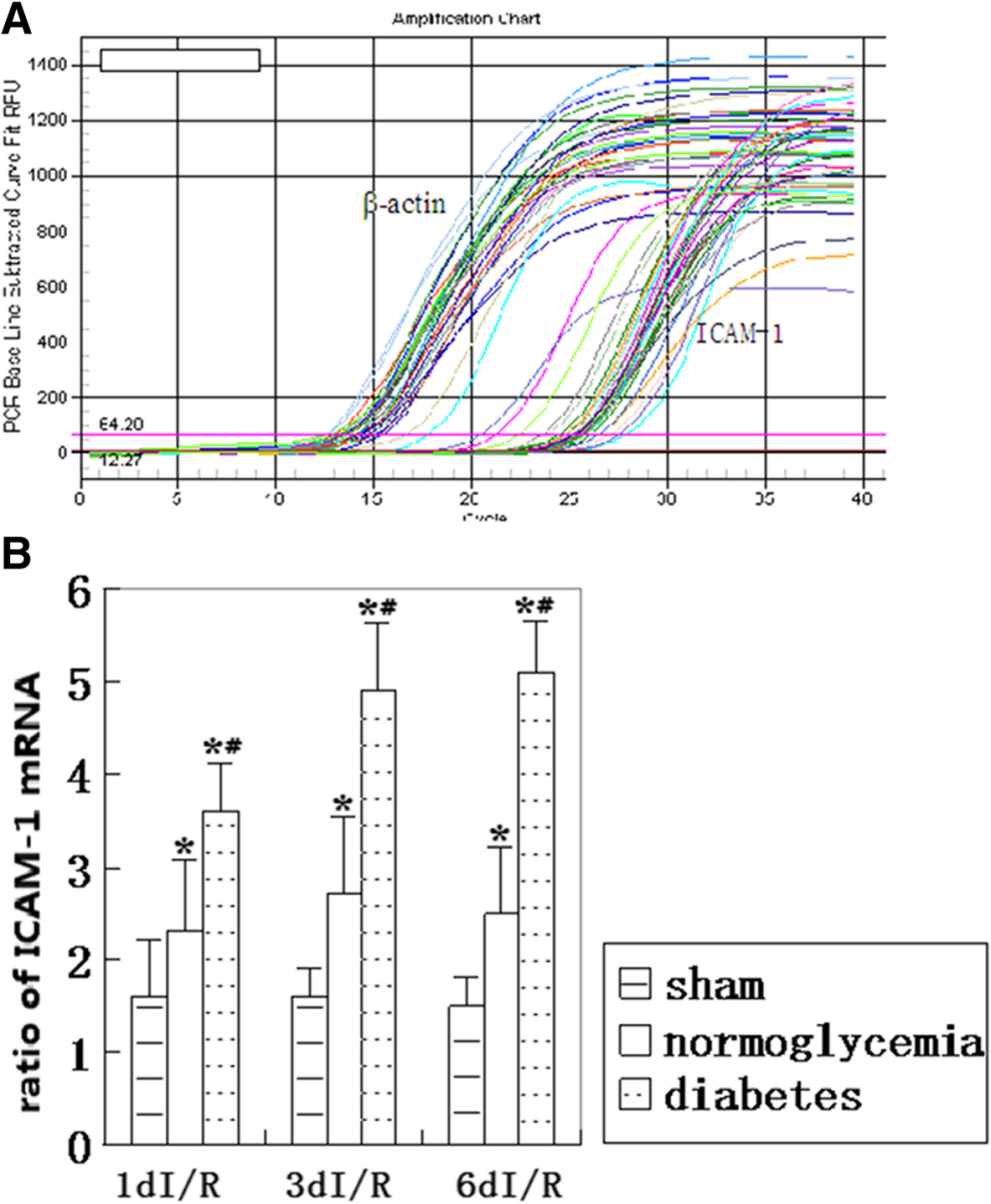
**ICAM-1 mRNA levels detected by RT-PCR. (A)** Examples of original RT-PCR recordings. **(B)** Bar graph summarizing ICAM-1 mRNA expression levels in the three groups. *P < 0.05 *vs*. sham group, #*P* < 0.05 *vs*. normoglycemic group at an identical reperfusion stage. I/R, ischemia and reperfusion.

ICAM-1 mRNA levels were detected by reverse transcription-polymerase chain reaction (RT-PCR). The Ct values (number of threshold cycle) of each foramen were recorded and compared. The results showed that the ICAM-1 mRNA levels were up-regulated after 1, 3, and 6 days of reperfusion in the normoglycemic rats and that diabetes further up-regulated the expression of the post-ischemic ICAM levels. The original RT-PCR recordings are presented in Figure 3A, and the summarized data are presented in Figure 3B.

### ICAM-1 protein level

**Figure 4:**
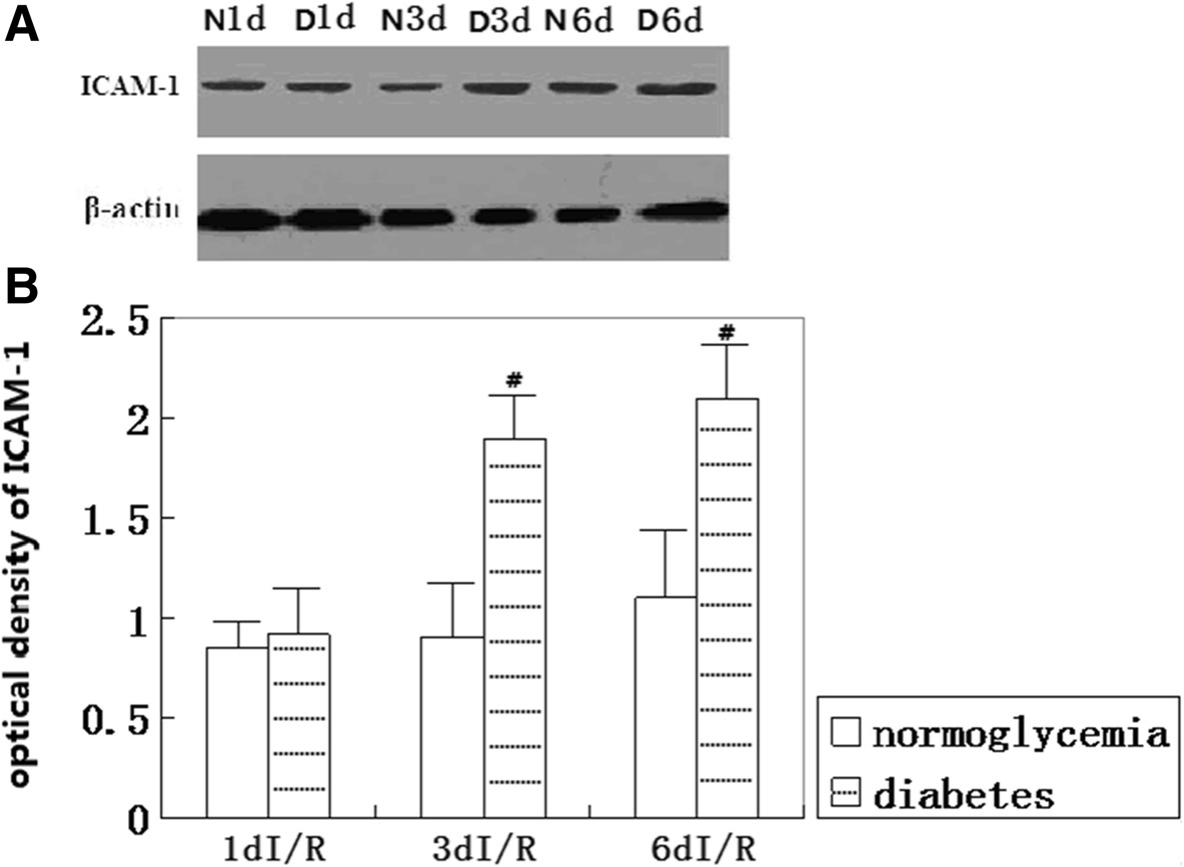
**ICAM-1 Western blotting. (A)** Western blot for ICAM-1. **(B)** Summarized bar graph indicating semi-quantitative levels of ICAM-1 protein in the normo- and hyperglycemic ischemic animals. N, normoglycemia; D, diabetes; and d, day. #*P* < 0.05 *vs*. normoglycemic group at an identical reperfusion stage.

The changes in the ICAM-1 protein levels in the frontal cortex after 8 min of cerebral ischemia in both the normoglycemic and diabetic hyperglycemic groups were analyzed by Western blotting. Band intensities were measured using the Bio-Rad gel-imaging system. As shown in Figure 4, compared to the normoglycemic animals, hyperglycemia significantly increased the ICAM-1 protein levels at 3 and 6 days of reperfusion in the frontal cortex.

### ICAM-1 mRNA *in situ* hybridization

**Figure 5:**
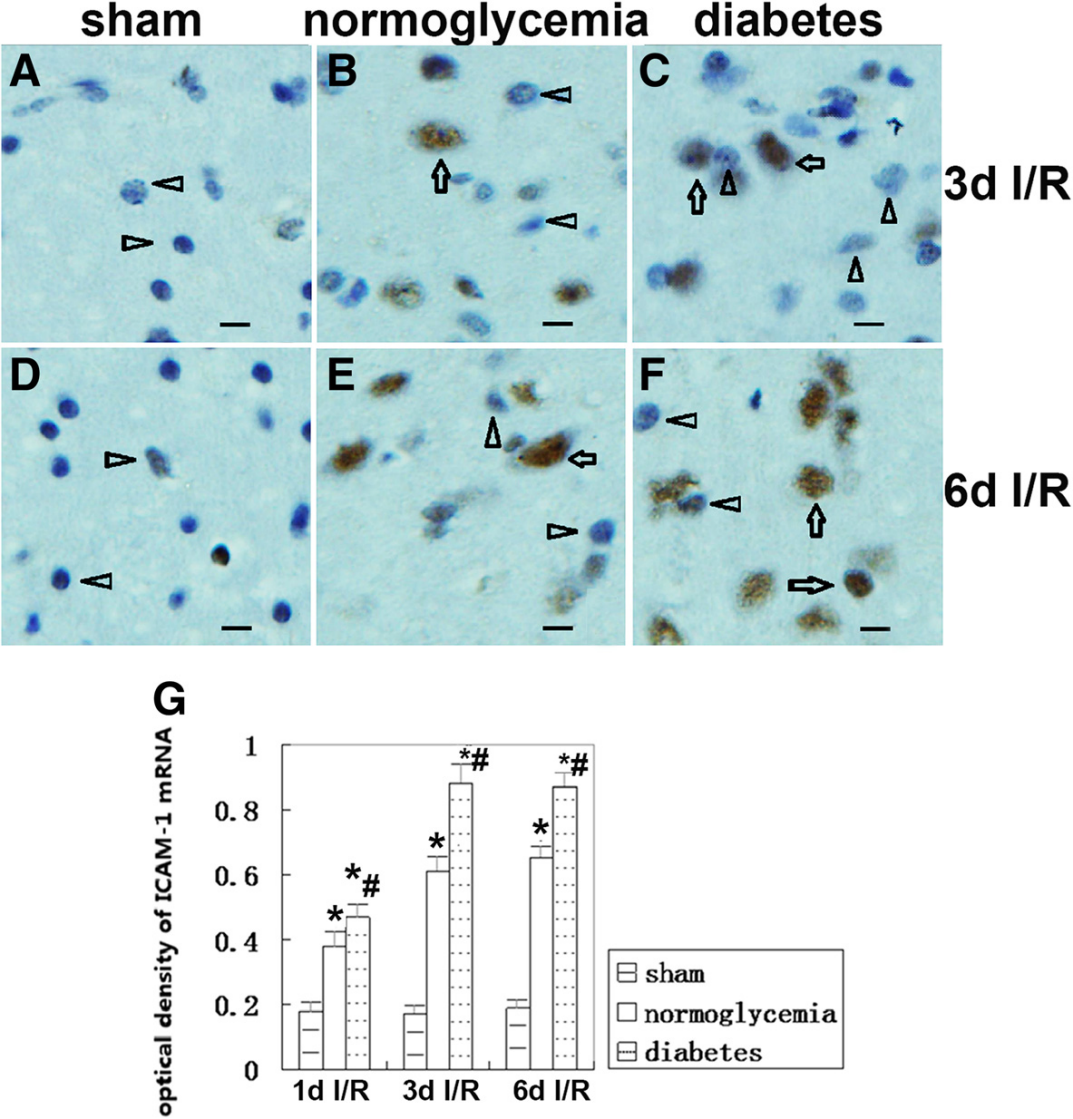
**ICAM-1 mRNA** ***in situ*** **hybridization.** Images were taken of the frontal cortex. **(A,D)** Negatively stained brain sections from sham controls. **(B,E)** ICAM-1 mRNA positive stained cells detected at 3 and 6 days of reperfusion in normoglycemic animals. **(C,F)** ICAM-1 mRNA positive stained cells in diabetic animals. **(G)** Bar graph summarizes the mean optical density of ICAM-1 mRNA in samples collected from the frontal cortex. Arrows indicate ICAM-1 positive neurons and arrow heads indicate ICAM-1 negative neurons. *P <0.05 *vs*. sham group; #*P* < 0.05 *vs*. normoglycemic group at an identical reperfusion stage. I/R, ischemia and reperfusion. DAB visualization, weak haematoxylin counterstaining. Magnification, 400X, bar = 20 μm.

Almost no ICAM-1 positive cells were detected in the frontal cortex of the sham control animals, and only the occasional few scattered positively stained neurons were detected. In contrast, the number of ICAM-1 mRNA positive cells was increased after 1 day and further increased after 3 days of reperfusion in normoglycemic rats subjected to 8 min of forebrain ischemia. Compared to the normoglycemic ischemic group, hyperglycemia increased the expression of ICAM-1 mRNA at 3 and 6 days of reperfusion. Examples of ICAM-1 *in situ* hybridization are shown in Figure 5A-F, and the summarized ICAM-1 mean optical density data [=integral optical density (IOD)/Sum area] are provided in Figure 5G.

### ICAM-1 cellular localization

**Figure 6:**
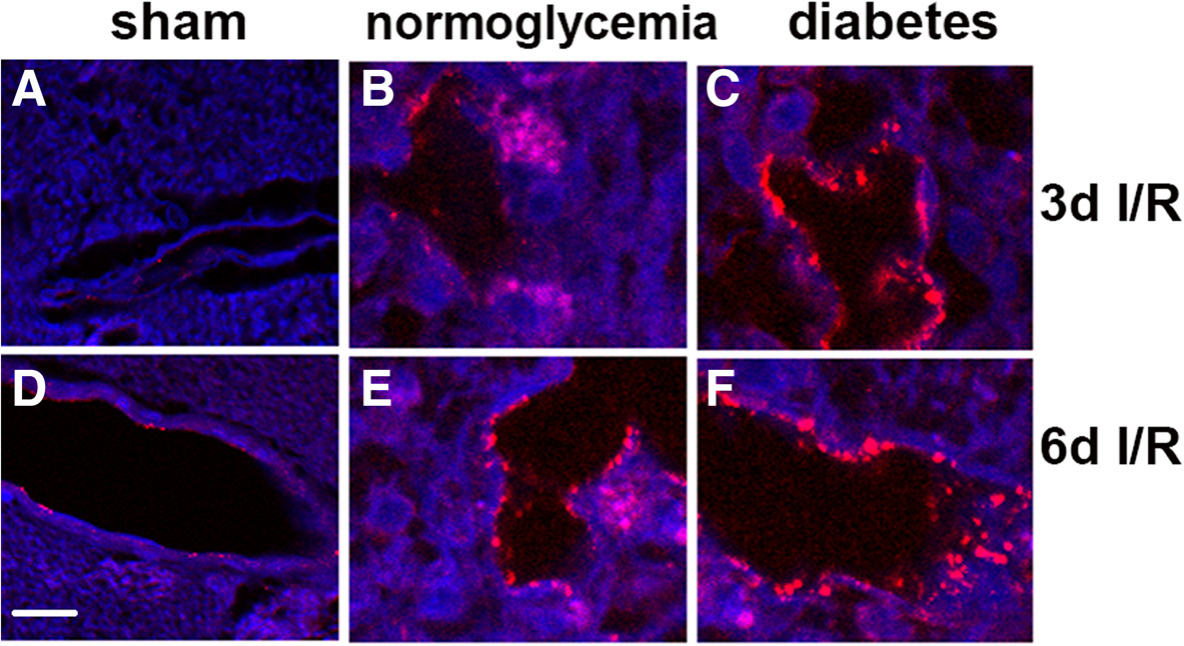
**ICAM-1 immunofluorescent staining (red color) in blood vessels of the frontal cortex. (A,D)** Sham group. **(B,E)** Normoglycemic ischemia after 3 and 6 days of reperfusion. **(C,F)** Hyperglycemic ischemia after 3 and 6 days of reperfusion. Red color, ICAM-1 staining, blue color, DAPI. Magnification, 400X.

ICAM-1 localization was detected using immunofluorescent staining in brain sections obtained from sham, normoglycemic ischemic and hyperglycemic ischemic animals. Weak ICAM-1 immunostaining was observed in the small blood vessels of the sham operated animal brain sections (Figure 6A and D). Eight minutes of cerebral ischemia induced the increased immunoreactivity of ICAM-1 in the small blood vessels of the normoglycemic animals at 3 and 6 days of reperfusion (Figure 6B and E). Diabetic ischemia further enhanced the ICAM-1 immunoreactivity in the cerebral blood vessels at 3 and 6 days of reperfusion (Figure 6C and F).

**Figure 7:**
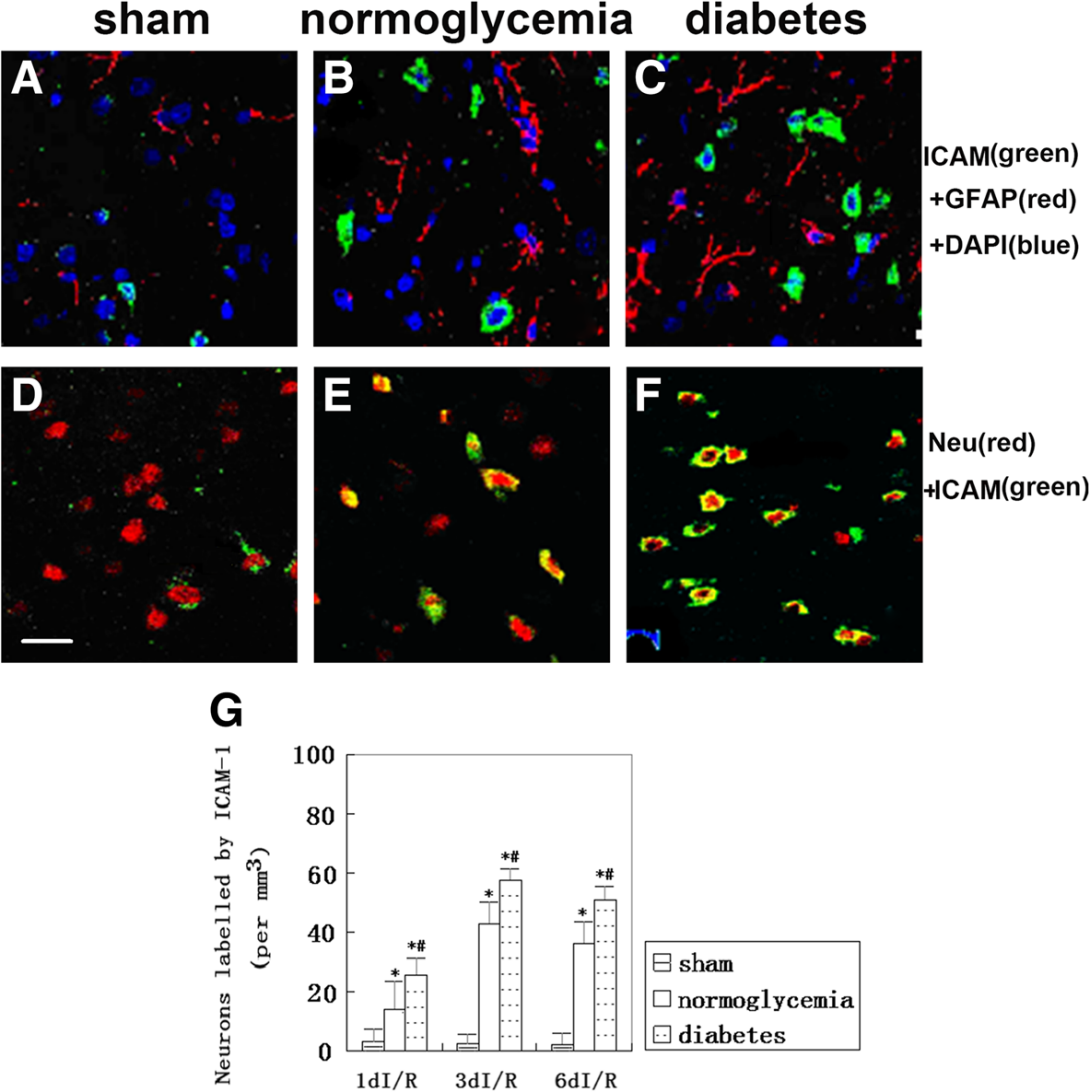
**Triple labeling of ICAM-1, GFAP and DAPI and double labeling of ICAM-1 and NeuN in the frontal cortex after 6 days of reperfusion in normo- and hyperglycemic animals. (A-C)** No colocalization of ICAM-1 with astrocytes; however, ischemia led to an increased number of GFAP positive astrocytes. **(D)** Occasional ICAM-1 positively stained neurons were detected in the sham control animals. **(E)** Ischemia increased the number of ICAM-1 positively stained neurons. **(F)** Diabetic hyperglycemia further increased the number of ICAM-1 positive neurons in the frontal cortex. **(G)** Bar graph summarizes the average number of neurons positively labeled by anti-ICAM1 antibody. *P <0.05 *vs*. sham group; #*P* < 0.05 *vs*. normoglycemic group at an identical reperfusion stage. Green color, ICAM-1; blue color, DAPI; red color, GFAP in **A**-**C** and NeuN in **D**-**F**. Magnification, 400X.

To examine whether ICAM-1 localized in other types of brain cells in addition to vascular endothelial cells, we performed double immune labeling of f ICAM-1 with the astrocyte marker GFAP, or the neuronal marker NeuN. The results showed that there was no colocalization of ICAM-1 with GFAP among the sham control, normoglycemic ischemic or hyperglycemic ischemic brain sections with up to 6 days of reperfusion (Figure 7A-C); however, both the numbers of GFAP-positive astrocytes and ICAM-1 positive cells increased independently after ischemia and reperfusion in both the normo- and hyperglycemic animals. In contrast, ICAM-1 colocalized with NeuN-positive neurons in the frontal cortex (Figure 7D-F). ICAM-1 neuronal immunoreactivity was occasionally observed in the sham-operated animals, increased in number and fluorescent intensity after 3 days of reperfusion following 8 min of ischemia, and were further aggravated by hyperglycemic ischemia and reperfusion.

## Discussion

It has been well established that diabetes/hyperglycemia exacerbates ischemic cerebral injury. Animal studies have confirmed that hyperglycemia enhances ischemia-induced brain edema, neuronal degeneration and mortality compared with normoglycemic animals [18]. In this study, we observed morphologic changes in the frontal cortex at 1, 3 and 6 days of reperfusion after 8 min of cerebral forebrain ischemia with or without diabetic hyperglycemia. Histological studies using H&E staining and Pischingert staining confirmed the exacerbation effects of hyperglycemia on neuronal death after ischemia, as reflected by the void spaces around neurons or vessels and the decreases in the Nissl bodies. Ideally, neurodegeneration should also be detected using FluoroJade staining and TUNEL staining, which is in addition to Pischingert staining. However, because neuronal damage in the hyperglycemic ischemic brain has been extensively studied and the main focus of the current study is ICAM, we believe a combination of Pischingert and H&E staining has satisfactorily revealed neuronal damage in the normo- and hyperglycemic animals. The factors that contribute to the adverse effects of hyperglycemia on the ischemic brain are not fully understood but may include tissue acidosis, the increased production of oxygen and nitrogen free radicals, damage to the mitochondria and the activation of neuro-inflammation.

ICAM-1 plays an important role in initiating neuro-inflammation after cerebral ischemia. ICAM-1, also known as CD54 (Cluster of Differentiation 54), is a protein that is encoded by the ICAM-1 gene in humans [20,21]. The ICAM-1 protein is continuously present in low concentrations in the membranes of leukocytes and endothelial cells. Upon cytokine stimulation, such as interleukin-1 (IL-1) and tumor necrosis factor (TNF) [22], the ICAM-1 concentration significantly increases, and it is expressed in the vascular endothelium, macrophages, and lymphocytes. Once activated, leukocytes bind to endothelial cells via ICAM-1/LFA-1 and then transmigrate into tissues [23], where it induces free radical production and the inflammatory cascade. Increased ICAM-1 further induces adhesion between vascular endothelial cells and leukocytes, impairs vascular endothelial cells and increases the permeability of capillaries, and eventually leads to brain damage [24,25]. It has been reported that the expression of ICAM-1 and the infiltration of neutrophils into ischemic tissue are closely correlated with the severity of ischemic brain damage [26]. The over-expression of ICAM-1 mRNA has been detected at 3 hours, peaks at 6 hours and persists to 5 days of reperfusion after middle cerebral artery occlusion [4], as well as after intracerebral hemorrhage. In the present study, we observed that ICAM-1 mRNA and protein levels dramatically increased in brain cortical tissues after 1, 3 and 6 days of recovery following 8 min of forebrain cerebral ischemia, suggesting ischemia induces inflammatory responses in the brain. We would like to note that the rat brain was not perfused with PBS because we wanted to extract brain tissue within 1 min of sacrifice to minimize the potential chemical changes induced by artificial post-complete ischemia due to extended intervals between decapitation and tissue harvest. This protocol might lead to an overestimation of ICAM-1 mRNA and protein levels due to residual neutrophils in the cerebral vessels. However, because the immunohistochemistry indicated a clear neuronal localization of ICAM-1, we predict the observed increases in ICAM-1 are largely due to an enhanced neuronal inflammatory response.

Diabetic hyperglycemia further enhanced the up-regulation of ICAM-1 induced by ischemia. It is known that hyperglycemia upregulates ICAM-1 in vascular endothelial cells [27–29]. Such an activation leads to inflammatory responses and to diabetic angiopathy and retinopathy. Acute hyperglycemia, induced by glucose infusion for 10 min prior to the induction of 10 min of forebrain ischemia, significantly increased the ICAM-1 protein levels in the brain homogenate and plasma together with elevations in superoxide radicals, malondialdehyde, high-mobility group box 1, and key markers of lipid peroxidation [30]. However, the sample collection time was only limited to up to 120 min following reperfusion. We have previously observed elevations of ICAM-1 and IL-1β after a very brief period of forebrain ischemia (5 min) in diabetic animals after 3 days of reperfusion [15]. In the present study, we examined the ICAM-1 transcription and translation levels, as well as ICAM-1 localization up to 6 days of reperfusion following a moderate period (8 min) of forebrain ischemia in normal and diabetic rats. The results revealed that diabetic hyperglycemia further increased the mRNA and protein levels of ICAM-1 in the frontal cortex after 1–6 days compared with the normoglycemic ischemic animals, suggesting that hyperglycemia aggravates ischemia-induced inflammation. The molecular mechanism by which hyperglycemia further enhances the expression of ICAM-1 is not fully understood. Previous studies have suggested that hyperglycemia may cause ICAM-1 increases through the activation of IL-1β and the p38 MAPK pathway [15,29].

To further explore whether ICAM-1 is expressed in cell types other than vascular endothelia, multiple immunolabelings of ICAM-1 with GFAP and NeuN were performed. The results revealed that ICAM-1 co-localized with cells other than astrocytes. The neuronal localization of ICAM-1 has been previously observed in healthy and inflamed choroid plexus [31] and dorsal root ganglia neurons after sciatic nerve injury [32]. In the same study, ICAM-1 immunoreactivity was also detected in Schwann and satellite cells. Our results demonstrate that ICAM-1 is localized not only in cerebrovascular endothelial cells but also in cortical neurons, implying diabetes leads to enhanced neuro-inflammation in post-ischemic brain tissues. The mechanisms through which hyperglycemia activates inflammatory responses are not fully understood. High glucose levels increase ROS accumulation due to the enhancement of ROS production and depletion of glutathione; the activation of the polyol, protein kinase C, MAPK pathways; and the enhanced production of advance glycation end-products (AGE). These events lead to the activation of NF-кB and activator protein 1 (AP-1) and the inhibition of nuclear factor (erythroid-derived 2)-like 2 (Nrf2). NF-кB is a potent inducer of inflammatory processes through its upregulation of the gene expression of proinflammatory cytokines and chemokines such as IL-1β, IL-6, IL-17, TNF-α, c-reactive proteins, chemo attractant protein 1, CCL-2 and CXC [33]. Nrf2 is a transcription factor that upregulates several antioxidant enzymes. In a recent study, Meng and colleagues demonstrated that diabetic hyperglycemia elicits neuro-inflammatory reactions through the activation of nucleotide-binding oligomerization domain (Nod) and Leucine-rich repeat containing protein 1 (NLRP1) [34].

## Conclusion

Diabetes mellitus leads to enhanced brain damage after cerebral ischemia. Diabetes further increases ischemia-induced ICAM-1 expression at the transcriptional and translational levels. Furthermore, ICAM-1 immunoreactivity was not only observed in vascular endothelial cells but also in neurons. The results suggest that increased neuro-inflammation may participate in mediating the diabetes-augmented brain damage caused by transient cerebral ischemia.

## Abbreviations

BBB: Blood– brain barrier
BCA: Bicinchoninic acid
CD54: Cluster of Differentiation 54
DAB: Diaminobezidine
DAPI: 4′, 6-diamidino-2-phenylindole
HRP: Horseradish peroxidase
ICAM-1: Intercellular cell adhesion molecule 1
GFAP: Glial fibrillary acidic protein
IOD: Integral optical density
IL-1: Interleukin-1
MAPK: Mitogen-activated protein kinase
OD: Optical density
PMN: Polymorphonuclear leukocyte
RT-PCR: Reverse transcription-polymerase chain reaction
STZ: Streptozotocin
TNF: Tumor necrosis factor

## References

[1] Mehta SL, Lin Y, Chen W, Yu F, Cao L, Li PA (2011). Manganese superoxide dismutase deficiency exacerbates ischemic brain damage under hyperglycemic conditions by altering autophagy. Transl Stroke Res.

[2] Supanc V, Biloglav Z, Kes VB, Demarin V (2011). Role of cell adhesion molecules in acute ischemic stroke. Ann Saudi Med.

[3] Krause GS, White BC, Aust SD, Nayini NR, Kumar K (1998). Brain cell death following ischemia and reperfusion: a proposed biochemical sequence. Crit Care Med.

[4] Wang X, Sirén AL, Liu Y, Yue TL, Barone FC, Feuerstein GZ (1994). Upregulation of intercellular adhesion molecule 1 (ICAM-1) on brain microvascular endothelial cells in rat ischemic cortex. Brain Res Mol Brain Res.

[5] Enzmann G, Mysiorek C, Gorina R, Cheng YJ, Ghavampour S, Hannocks MJ, Prinz V, Dirnagl U, Endres M, Prinz M, Beschorner R, Harter PN, Mittelbronn M, Engelhardt B, Sorokin L (2013). The neurovascular unit as a selective barrier to polymorphonuclear granulocyte (PMN) infiltration into the brain after ischemic injury. Acta Neuropathol.

[6] Choi JS, Park J, Suk K, Moon C, Park YK, Han HS (2011). Mild hypothermia attenuates intercellular adhesion molecule-1 induction via activation of extracellular signal-regulated kinase-1/2 in a focal cerebral ischemia model. Stroke Res Treat.

[7] Fotis L, Agrogiannis G, Vlachos IS, Pantopoulou A, Margoni A, Kostaki M, Verikokos C, Tzivras D, Mikhailidis DP, Perrea D (2012). Intercellular adhesion molecule (ICAM)-1 and vascular cell adhesion molecule (VCAM)-1 at the early stages of atherosclerosis in a rat model. In Vivo.

[8] Zhu YP, Shen T, Lin YJ, Chen BD, Ruan Y, Cao Y, Qiao Y, Man Y, Wang S, Li J (2013). Astragalus polysaccharides suppress ICAM-1 and VCAM-1 expression in TNF-α-treated human vascular endothelial cells by blocking NF-κB activation. Acta Pharmacol Sin.

[9] Li M, Qu YZ, Zhao ZW, Wu SX, Liu YY, Wei XY, Gao L, Gao GD (2012). Astragaloside IV protects against focal cerebral ischemia/reperfusion injury correlating to suppression of neutrophils adhesion-related molecules. Neurochem Int.

[10] Su Y, Mao N, Li M, Dong X, Lin FZ, Xu Y, Li YB (2013). Sarpogrelate inhibits the expression of ICAM-1 and monocyte–endothelial adhesion induced by high glucose in human endothelial cells. Mol Cell Biochem.

[11] Guan T, Liu Q, Qian Y, Yang H, Kong J, Kou J, Yu B (2013). Ruscogenin reduces cerebral ischemic injury via NF-κB-mediated inflammatory pathway in the mouse model of experimental stroke. Eur J Pharmacol.

[12] Ge H, Wen Y, Yang G, Betz AL (2000). Increased expression of intercellular adhesion molecule-1 in mouse focal cerebral ischemia model. Chin Med J (Engl).

[13] Cybulsky MI, Iiyama K, Li H, Zhu S, Chen M, Iiyama M, Davis V, Gutierrez-Ramos JC, Connelly PW, Milstone DS (2001). A major role for VCAM-1, but not ICAM-1, in early atherosclerosis. J Clin Invest.

[14] Navarro JF, Mora C (2005). Role of inflammation in diabetic complications. Nephrol Dial Transplant.

[15] Ding C, He Q, Li PA (2005). Diabetes increases expression of ICAM after a brief period of cerebral ischemia. J Neuroimmunol.

[16] Smolock AR, Mishra G, Eguchi K, Equchi S, Scalia R (2011). Protein kinase C upregulates intercellular adhesion molecule-1 and leukocyte-endothelium interactions in hyperglycemia via activation of endothelial expressed calpain. Arterioscler Thromb Vasc Biol.

[17] Li PA, Shamloo M, Katsura K, Smith ML, Siesjo BK (1995). Critical values for plasma glucose in aggravating ischaemic brain damage: correlation to extracellular pH. Neurobiol Dis.

[18] Jing L, Zhang JZ, Zhao L, Wang YL, Guo FY (2007). High-expression of transforming growth factor β1 and phosphorylation of extracellular signal-regulated protein kinase in vascular smooth muscle cells from aorta and renal arterioles of spontaneous hypertension rats. Clin Exp Hypertens.

[19] Jing L, Zhang JZ, Sun JP, Guo FY, An X, Yang K, Li PA (2011). Inhibition of extracellular sinal-regulated kinases amerliorates hypertension-induced renal vascular remodeling in rat models. Int J Mol Sci.

[20] Carlson M, Nakamura Y, Payson R, O’Connell, Leppert M, Lathrop GM, Lalouel JM, White R (1988). Isolation and mapping of a polymorphic DNA sequence (pMCT108. 2) on chromosome 18 [D18S24]. Nucleic Acids Res.

[21] Katz FE, Parkar M, Stanley K, Murray LJ, Clark EA, Greaves MF (1985). Chromosome mapping of cell membrane antigens expressed on activated B cells. Eur J Immunol.

[22] Nie Z, Fryer AD, Jacoby DB (2012). β2-Agonists inhibit TNF-α-induced ICAM-1 expression in human airway parasympathetic neurons. PLoS One.

[23] Yang L, Froio RM, Sciuto TE, Dvorak AM, Alon R, Luscinskas FW (2005). ICAM-1 regulates neutrophil adhesion and transcellular migration of TNF-α-activated vascular endothelium under flow. Blood.

[24] Yilmaz G, Granger DN (2010). Leukocyte recruitment and ischemic brain injury. Neuromolecular Med.

[25] Cao J, Shi X, Li W, Liu J, Miao X, Xiu J (2009). Protective effect of anti-intercellular adhesion molecule-1 antibody on global cerebral ischemia/reperfusion injury in the rat. Biosci Trends.

[26] Liu L, Wang Z, Wang X, Song L, Chen H, Bémeur C, Ste-Marie L, Montgomery J (2007). Comparison of two rat models of cerebral ischemia under hyperglycemic conditions. Microsurgery.

[27] Bačun T, Glavaš-Obrovac L, Belovari T, Mihaljević I, Hanich T, Belaj VF, Vcev A (2010). Insulin administration in the mild hyperglycaemia changes expression of proinflammatory adhesion molecules on human aortic endothelial cells. Coll Antropol.

[28] Kern TS (2007). Contributions of inflammatory processes to the development of the early stages of diabetic retinopathy. Exp Diabetes Res.

[29] Kim SW, Kim CE, Kim MH (2011). Flavonoids inhibit high glucose-induced up-regulation of ICAM-1 via the p38 MAPK pathway in human vein endothelial cells. Biochem Biophys Res Commun.

[30] Tsuruta R, Fujita M, Ono T, Koda Y, Koga Y, Yamamoto T, Nanba M, Shitara M, Kasaoka S, Maruyama I, Yuasa M, Maekawa T (2010). Hyperglycemia enhances excessive superoxide anion radical generation, oxidative stress, early inflammation, and endothelial injury in forebrain ischemia/reperfusion rats. Brain Res.

[31] Wolburg K, Gerhardt H, Schulz M, Wolburg H, Engelhardt B (1999). Ultrastructural localization of adhesion molecules in the healthy and inflamed choroid plexus of the mouse. Cell Tissue Res.

[32] Yang J, Gu Y, Huang X, Shen A, Cheng C (2011). Dynamic changes of ICAM-1 expression in peripheral nervous system following sciatic nerve injury. Neurol Res.

[33] Sandireddy R, Yerra VG, Areti A, Komirshetty P, Kumar A (2014). Neuroinflammation and oxidative stress in diabetic neuropathy: futuristic strategies based on these targets. Int J Endocrinol.

[34] Meng X-F, Wang X-L, Tian X-J, Yang Z-H, Chu G-P, Zhang J, Li M, Shi J, Zhang C (2014). Nod-like receptor protein 1 inflammasome mediates neuron injury under high glucose. Mol Neurobiol.

